# Adult tissue–derived neural crest‐like stem cells: Sources, regulatory networks, and translational potential

**DOI:** 10.1002/sctm.19-0173

**Published:** 2019-11-18

**Authors:** Pihu Mehrotra, Georgios Tseropoulos, Marianne E. Bronner, Stelios T. Andreadis

**Affiliations:** ^1^ Department of Chemical and Biological Engineering University at Buffalo Buffalo New York; ^2^ Division of Biology and Biological Engineering California Institute of Technology Pasadena California; ^3^ Center of Excellence in Bioinformatics and Life Sciences Buffalo New York; ^4^ Department of Biomedical Engineering University at Buffalo Buffalo New York

**Keywords:** demyelinating disorders, gene regulatory network, neural crest, Schwann cells

## Abstract

Neural crest (NC) cells are a multipotent stem cell population that give rise to a diverse array of cell types in the body, including peripheral neurons, Schwann cells (SC), craniofacial cartilage and bone, smooth muscle cells, and melanocytes. NC formation and differentiation into specific lineages takes place in response to a set of highly regulated signaling and transcriptional events within the neural plate border. Premigratory NC cells initially are contained within the dorsal neural tube from which they subsequently emigrate, migrating to often distant sites in the periphery. Following their migration and differentiation, some NC‐like cells persist in adult tissues in a nascent multipotent state, making them potential candidates for autologous cell therapy. This review discusses the gene regulatory network responsible for NC development and maintenance of multipotency. We summarize the genes and signaling pathways that have been implicated in the differentiation of a postmigratory NC into mature myelinating SC. We elaborate on the signals and transcription factors involved in the acquisition of immature SC fate, axonal sorting of unmyelinated neuronal axons, and finally the path toward mature myelinating SC, which envelope axons within myelin sheaths, facilitating electrical signal propagation. The gene regulatory events guiding development of SC in vivo provides insights into means for differentiating NC‐like cells from adult human tissues into functional SC, which have the potential to provide autologous cell sources for the treatment of demyelinating and neurodegenerative disorders.

## Graphical abstract

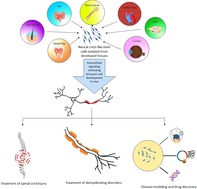

**Significance statement**

Neural crest (NC) cells have attracted attention for their multipotent nature and ease of isolation from adult tissues. This concise review reports the advantages of using NCs for the treatment of demyelinating disorders and spinal cord injury (SCI), over other cell sources such as induced pluripotent stem cells and embryonic stem cells. Adult tissue‐derived NCs are easy to expand in vitro and can be derived from autologous sources. Moreover, differentiation of NCs to Schwann cells (SC) can be easily achieved without genetic mutation, making them safe for translation from a laboratory to a clinical setting. Adult NC‐derived SC are functional and can myelinate neurons in vitro and spinal cord in vivo in mice. Hence, NCs derived from adult tissue are a promising cell source for the treatment of demyelinating disorders and SCI. Furthermore, this technology can also be used for disease modeling and drug testing, making way for personalized therapeutics for neurological disorders.

## 1 INTRODUCTION TO THE NEURAL CREST CELLS

Often referred to as the “fourth germ layer,” the neural crest (NC) is a multipotent and migratory stem cell population that contributes to a wide array of organs and tissues in the vertebrate embryo, including autonomic ganglia, sensory neurons, adrenal and thyroid glands, cartilage and bone of the face, smooth muscle cells of some major arteries, and melanocytes in the skin.^1^, ^2^ NC formation is first observed at stage 9 of human embryogenesis and extends till stage 20 as per the Carnegie staging system.^3^ Neural crest stem cells (NCSC) were first identified in rodents by Stemple and Anderson and isolated using cell sorting for NC‐specific cell surface protein p75^NTR^ (neurotrophin receptor [NTR]). These p75^NTR+^ cells could self‐renew and generate neurons and glia of the PNS as well as myofibroblasts.^4^ The high degree of self‐renewal and regenerative capacity of NC makes it a very attractive source for stem cell‐based therapies.

During embryonic development, formation of NC stem cells originates in response to a set of signaling events between neural and non‐neural ectoderm, a region termed as the neural plate border.^5^ Induction is initiated at the plate border by signals including fibroblast growth factor (FGF), bone morphogenetic protein (BMP), and Wnt.^5^, ^6^ On establishment of the neural plate border territory, new signaling events are established for specification of bona fide NCSCs. This process results in expression of NC specifiers (Snail2, Sox8, Sox9, Sox10, FoxD3, c‐Myc, and Id family members) that in turn mediate changes in shape, motility, and adhesive properties leading to delamination of NCSCs from the neuroepithelium and initiating cell lineage decisions.^6^ NC precursor cells within the dorsal neural tube then undergo epithelial to mesenchymal transition and migrate along precise pathways,^7^ eventually settling and differentiating into specialized cell types, according to their axial origin as well as environmental cues encountered during the process.^8^ Cranial NCSCs form craniofacial structures of the head including cartilage and bone tissue of the skull and face, as well as cranial neurons, glia, and connective tissue of the face. Trunk NCSCs differentiate into dorsal root ganglia (DRG), containing sensory neurons and satellite glial cells, endocrine cells of the adrenal glands, and Schwann cells (SC) along the spinal nerves. Some of these NCSCs also differentiate into melanocytes in the skin. Finally, vagal NCSCs populate the enteric nervous system (ENS) along the length of the gut and contribute to the connective tissues of the arteries, septation of the outflow tract and cardiac ganglion.^1^

## 2 SOURCES OF NC‐LIKE STEM CELLS IN ADULT TISSUES

### 2.1 NC characteristics are retained postembryonically in adult tissues

Although NC cells are often referred to as stem or stem‐like cells, they are a transient population in the embryo that becomes progressively restricted in developmental potential during the course of development and rapidly lose their multipotency. Nevertheless, recent evidence suggests that many NC derivatives, including skin, cornea, gut, and peripheral nerves, contain stem or precursor cells with the ability to give rise to multiple NC derivatives.^1^, ^9^ These tissues provide potential sources for obtaining multipotent neural crest‐like stem cells (NClSCs) for the purpose of regenerative medicine and cell therapy, and consequently, they are under study in several laboratories. Whether NC‐like cells from the adult tissue come from embryonic NCs and remain dormant in a multipotent state or they attain a specialized fate and dedifferentiate to their NC progeny in specific culture conditions has not been established yet. The answers to these questions can only be attained by lineage‐tracing experiments tracking migration and differentiation of NCs starting from embryo development and throughout adulthood.

NClSCs have been isolated from mouse bone marrow and DRG. Nagoshi et al used double‐transgenic mouse strains P0 and Wnt1‐Cre/Floxed‐EGFP to map and isolate NClSCs from bone marrow, DRG, and whisker pad using cell‐sorting. P0 and Wnt1 are marker genes expressed in NC cells in mouse embryos.^10^ Interestingly, DRG‐derived NClSCs generated more primary and secondary spheres compared with bone marrow and whisker pad–derived cells and showed increased expression of NC markers p75NTR, Sox10, Nestin, and Musashi1. Additionally, 75% of DRG‐derived spheres showed tri‐lineage differentiation to neurons, glia, and myofibroblasts, compared with only 7.3% of spheres derived from whisker pad and 3.3% in spheres derived from bone marrow.^10^ These results highlight the tissue‐dependent differences in the abundance and self‐renewal capacity of adult NClSCs, which may ultimately determine the suitability for cellular therapies. Furthermore, bone marrow–derived NCs could be differentiated into SC that showed myelination around DRG neuronal axons in vitro, indicating their potential for use in peripheral nerve regeneration.^11^

During development, NCs also migrate to the cornea, where their derivatives remain throughout adulthood. The cornea is a transparent avascular structure that covers the front of the eye and helps to focus light on the retina. The cornea consists of three major cellular components: a stratified epithelium, a collagenous stroma containing keratocytes, and a layer of endothelium.^1^ Experiments with chick‐quail chimeras demonstrated that cranial NC cells form the corneal endothelium and keratocytes.^12^, ^13^, ^14^, ^15^ Lineage tracing experiments demonstrated that upon transplantation from late into early chick embryos quail NC‐derived keratocytes followed normal migratory routes giving rise to smooth muscle cells, myofibrils, keratocytes, and endothelial cells but failed to differentiate into neurons or cartilage, suggesting restricted plasticity of these NC‐derived progenitors.^16^ Another study however demonstrated differentiation of corneal progenitor cells into keratocytes, fibroblasts, myofibroblasts, adipocytes, chondrocytes, and neural cells, indicating similar differentiation potential to NCSC.^17^

Stem cell derivatives of NC origin persist in the adult rodent gut from which they can be isolated based on CD49b expression. Sorted cells showed expression of NC marker genes, such as p75NTR, Sox10, and Nestin, along with markers specific to enteric glia such as S100B and glial fibrillary acidic protein.^18^ Multipotent progenitors from fetal gut exhibited more plasticity and degree of self‐renewal compared with those derived from adult gut. In addition, upon transplantation into chick embryos, fetal progenitors gave rise primarily to neurons, whereas postnatal gut progenitors turned predominantly into glia.^19^ In vivo grafting experiments suggest therapeutic potential of these progenitors for the treatment of ENS disorders.^20^, ^21^

Cardiac NC cells play a role in septation of cardiac outflow tract into pulmonary and aortic branches.^1^ In vitro clonal expansion of NCISCs isolated from cardiac tissue suggested that only a small fraction of them were capable of self‐renewal and generation of NC derivatives.^22^ Stem cells from neonatal rodent heart demonstrate a “NC‐like” behavior, and when grown as cardiospheres, they could differentiate into PNS neurons, glia, smooth muscle cells, and cardiomyocytes, as well as migrate to tissues characteristic for NC derivatives in ovo.^23^ Interestingly, cardiac *resident Nestin+* progenitors migrated to areas damaged by infarction and contributed to reparative vascularization, indicating their potential for the treatment of various heart diseases.^24^

Although the presence of NClSCs in developed tissues provides evidence of an alternative cell source for cell therapy, most studies of this type have been limited to rodents, due to inaccessibility of human NCs in organs such as gut, heart, DRG and spine, as shown in Figure S1. Interestingly though, NCISCs have also been isolated from adult human tissues such as skin and dental pulp. The isolation of NClSCs from skin tissue enhances their therapeutic potential, mostly because of the accessibility and size of skin tissue that can provide autologous cells for cell therapies.^25^, ^26^, ^27^, ^28^ Fernandes et al showed that endogenous adult dermal precursors residing in the papillae of hair and whisker follicles could give rise to multipotent NClSCs.^25^ Similarly, Sieber‐Blum et al reported the presence of multipotent NCISCs in the adult mammalian hair follicle, which could give rise to neurons, melanocytes, smooth muscle cells, SC, and chondrocytes in vitro. These multipotent cells were termed epidermal neural‐crest cells,^26^ and when grafted in a mouse model of SCI, they integrated into the host spinal tissue yielding improvements in touch perception and sensory connectivity.^29^

### 2.2 Multipotent NClSCs from interfollicular epidermis

Recently, in our laboratory, Bajpai et al devised a method to reprogram postnatal human epidermal keratinocytes (KCs) to NClSC (termed KC‐NC) by mimicking signaling events that occur at the neural plate border. Transcriptomic analysis confirmed that epidermally derived NCISCs were similar to those generated from human embryonic stem cells (ESCs) and maintained the multilineage differentiation potential into melanocytes, neurons, SC, and mesenchymal cells in vitro and in ovo.^30^ In a subsequent study, we identified the factors that promote expansion of KC‐NC and maintain the NC phenotype. Specifically, we showed that FGF2 was necessary and sufficient for expression of Sox10, but both FGF2 and IGF1 worked synergistically to upregulate FoxD3. In addition, inhibition of TGF‐β1 further enhanced Sox10 expression.^31^ We also demonstrated that the same signaling factors can be used to obtain multipotent and functional NClSCs from cultures of human inter‐follicular KC isolated from elderly donors.^32^ Interestingly, NClSC from older donors exhibited significantly younger epigenetic age than epidermal KC, perhaps indicating greater potential for cell therapies. Given the accessibility, high proliferative capacity, and ease of reprogramming without genetic modification, KC‐NC represent an abundant, autologous source of functional therapeutic cells for regenerative medicine. They can also provide an excellent culture system for studying human disease, similar to induced pluripotent stem cells (iPSCs) but without the need for genetic modification or reprogramming to the pluripotent state.

### 2.3 Schwann cell precursors contribute to NC derivatives

Recent evidence suggests that NC cells that become associated with peripheral nerves acquire a partial glial phenotype, assuming the characteristics of a “Schwann cell precursor” (SCP). Intriguingly, it has been shown that late embryonic stages, many melanocytes originate from nerve‐associated SCPs.^33^ This occurs well after NC cells destined to form melanocytes have emigrated from the neural tube. Similarly, lineage analysis in mice has shown that cranial parasympathetic ganglia as well as a subpopulation of enteric neurons arise from this cell population.^34^, ^35^, ^36^ This is consistent with the possibility that later NC derivatives may arise from an SCP population that represents a nascent stem cell population associated with peripheral nerves. These SCPs express characteristic marker genes that are different from those expressed by migratory NCSC but similar to markers expressed by immature SC. For example, SCPs express genes encoding myelin basic protein (MBP), peripheral myelin protein 22 (PMP22), desert hedgehog, Cadherin 19, Connexin 29, GAP43, BFABP, and other Schwann cell markers, many of which are also associated with differentiation into myelinating SC.^37^, ^38^, ^39^, ^40^, ^41^ However, rather than being restricted to differentiate into SC, SCPs remain multipotent and appear to have the ability to contribute to numerous lineages at much later times in embryogenesis than normally associated with NC migration. In fact, recent studies also suggest that SCPs may give rise to chromaffin cells of the adrenal medulla, as evidenced by single cell RNA sequencing (scRNA‐seq) of the developing adrenomedullary cells in mice.^42^ This raises the intriguing possibility that these may represent true stem cells with remarkable multipotency and regenerative ability.

The transition from migratory NC cell to SCPs likely occurs when NC cells approach and/or become associated with peripheral nerves emanating from sensory and autonomic ganglia. At this point, the NC‐derived cells upregulate genes typically associated with SC while downregulating more typical NC markers, thus assuming a more glial like state.^38^, ^43^ Although these cells may be biased toward glial lineages, they remain multipotent and we speculate that perhaps they “dedifferentiate” similar and give rise to other NC fates similar to radial glia which are neuronal progenitors in the central nervous system. Whether SCPs maintain their multipotency into adulthood and the full range of cell types into which they can differentiate remain open questions.

## 3 GENE REGULATION IN NC CELLS: FATE ACQUISITION AND MULTIPOTENCY MAINTENANCE

### 3.1 An NC gene regulatory network controls lineage diversification into neuronal, melanocytic, and glial, including Schwann cell lineages

The NC is an excellent model system for studying questions of stem cell biology due to its multipotency, motility, and ability to form a broad array of derivatives. These are as diverse as neurons and SC of the peripheral nervous system, craniofacial cartilage, and bone, as well as skin melanocytes. These inherent stem cell properties have potentially important implications in regenerative medicine to treat disorders like familial dysautonomia, cleft palate, and NC‐related heart conditions such as Persistent Truncus Arteriosus, as well as to understand anomalies in differentiation that lead to cancers such as melanomas and Schwannomas.

The recent expansion of molecular biological techniques including single‐cell RNA‐seq, ChIP‐seq, and ATAC‐seq have facilitated the dissection of the genetic program controlling NC development. These genomic approaches have provided important insights into gene regulatory mechanisms and uncovered new regulatory factors involved in control of NC formation and diversification.^44^, ^45^ It is now clear that an intricate array of transcription factors and signaling molecules act in concert to imbue NC cells with its broad multipotency and migratory ability. These factors have been proposed to act via a multistep NC gene regulatory network (GRN) that integrates transcriptional inputs and diverse environmental signals.^2^, ^5^, ^46^, ^47^ The NC GRN consists of a series of hierarchically arranged regulatory steps, including induction of the prospective NC at the neural plate border, specification of multipotent NC cells within the dorsal neural tube, control of their delamination via an epithelial to mesenchymal transition to produce a migratory population, and finally, diversification into distinct cell lineages.

The NC GRN posits that the process of NC formation is comprised of a logical series of distinct regulatory steps that flow seamlessly from one to the other. First, signaling molecules, including Wnts, FGFs, and BMPs, in the gastrula stage embryo initiate the process of NC induction by inducing transcription factors like *Msx*, *Pax3/7*, *Zic1*, and *Dlx3/5* at the border between the neural plate and nonneural ectoderm. The region where these genes are coexpressed defines the neural plate border—a domain primed to form bona fide NC cells. These in turn function in combination with signaling molecules to regulate “NC specifier genes” like *Snail/Slug*, *AP‐2*, *FoxD3*, *Twist*, *Id*, *cMyc*, and *Sox8/9/10*. The NC GRN itself can be envisioned as a series of sequential binary decisions leading to differentiation into derivative fates. Recently *Twist1* has been reported to play a critical role as a regulator of NC fate decision, and it biases NC commitment toward a mesenchymal fate.^45^ Conversely, *FoxD3* represses the mesenchymal program of delaminating NCs.^48^ The postmigratory program begins following the downregulation of transcription factors associated with the neural tube program such as *Zic3* and *Pax8*.

Expression of the Sox family genes initiates within the dorsal neural tube and defines cells with the potential to emigrate from the neural tube and form migratory NC cells. In particular, the SoxE transcription factors (Sox8/9/10) are critical regulators of most NC lineages.^5^, ^49^, ^50^ Specifically, Sox8 and Sox9 are expressed early in the newly induced NC, preceding Sox10, which serves as a nearly pan migratory NC marker. At later developmental stages, Sox9 and Sox10 persist in specific NC subpopulations. Whereas Sox9 is maintained in NC‐derived chondrocytes, Sox10 persists in neuronal, glial, and melanocyte lineages and controls their specification in combination with different cofactors in each lineage.

Sox10 regulates differentiation of sensory and autonomic lineages by regulating expression of achaete‐scute homolog 1 and the paired homeodomain (Phox2b) transcription factors that are essential for neurogenesis in the autonomic lineage.^51^ In the DRG, transient expression of Sox10 regulates expression of neurogenin.^52^ Sox10 also binds to endothelin receptor‐B (EDNRB) to regulate development of the ENS, and disruption in this binding has been shown to cause Hirschsprung disease.^53^ Sox10 also is critical for emergence of the glial lineage by regulating Oct6 and Krox20 (Egr2) transcription factors, which are critical for myelination.^54^, ^55^, ^56^ Its expression persists through subsequent stages of terminal differentiation,^49^ regulating expression of myelin proteins, including protein zero (P0)^57^, MBP, PMP22, and the gap junction protein connexin 32.^58^

### 3.2 Diverse signaling pathways govern NC cell fate during embryogenesis

Many studies have focused on discovering the regulatory pathways that control NC fate acquisition in order to better understand cell fate specification during embryonic development in vivo and develop bioinspired strategies to differentiate NC into different lineages in vitro.

After formation of the neural tube, NC cells separate from the neuroepithelium and migrate to distant anatomic locations, while making cell lineage decisions in response to multiple morphogenetic signals.^59^, ^60^ However, some NCs remain unspecified and retain their stemness and multipotency.^9^, ^61^ In general, there is significant heterogeneity with respect to the differentiation capacity of NCs, with some giving rise to multiple derivatives while others differentiating into a subset of cell types,^62^, ^63^ depending on anatomic location and the presence of fate specifying signals. NCs from all axial levels give rise to neurons, glia, and pigment cells, but mesenchymal cell specification depends on axial position. Cranial NCs give rise to skeletal mesenchyme; trunk NCs give rise to dorsal fin mesenchyme in amphibians and fish; and vagal NCs contribute to smooth muscle cells of the cardiac outflow tract.^64^, ^65^

One of the earliest NC markers includes the family of receptor tyrosine kinases (RTK), which plays an important role in cell migration and survival. The RTK family are critical for development of NC derivatives in vivo.^66^ At migratory stages, the neurotrophin factor NT‐3 binds to the trkC receptor and induces sensory neurogenesis.^67^ GDNF binds to the RET receptor during ENS development.^66^ Neuregulin binds to receptor ErbB3 and is required for glia formation in the peripheral nervous system.^68^ Binding of steel factor to the c‐kit receptor promotes formation of melanocyte precursors,^60^ whereas cartilage precursors express platelet‐derived growth factor receptor α.^69^

The timing of NC migration during development also affects the fate they attain as cell specific genes are expressed at different times during migration. Early‐migrating cells express transcription factors Brn3 or neurogenin‐2 and commit to sensory neuron lineage.^70^, ^71^ Late‐migrating cells express melanocytic‐specific markers in the migration staging area after departure of the early‐migrating NCs.^72^ These signals contribute to the molecular heterogeneity of the premigratory NCs.

**Figure 1:**
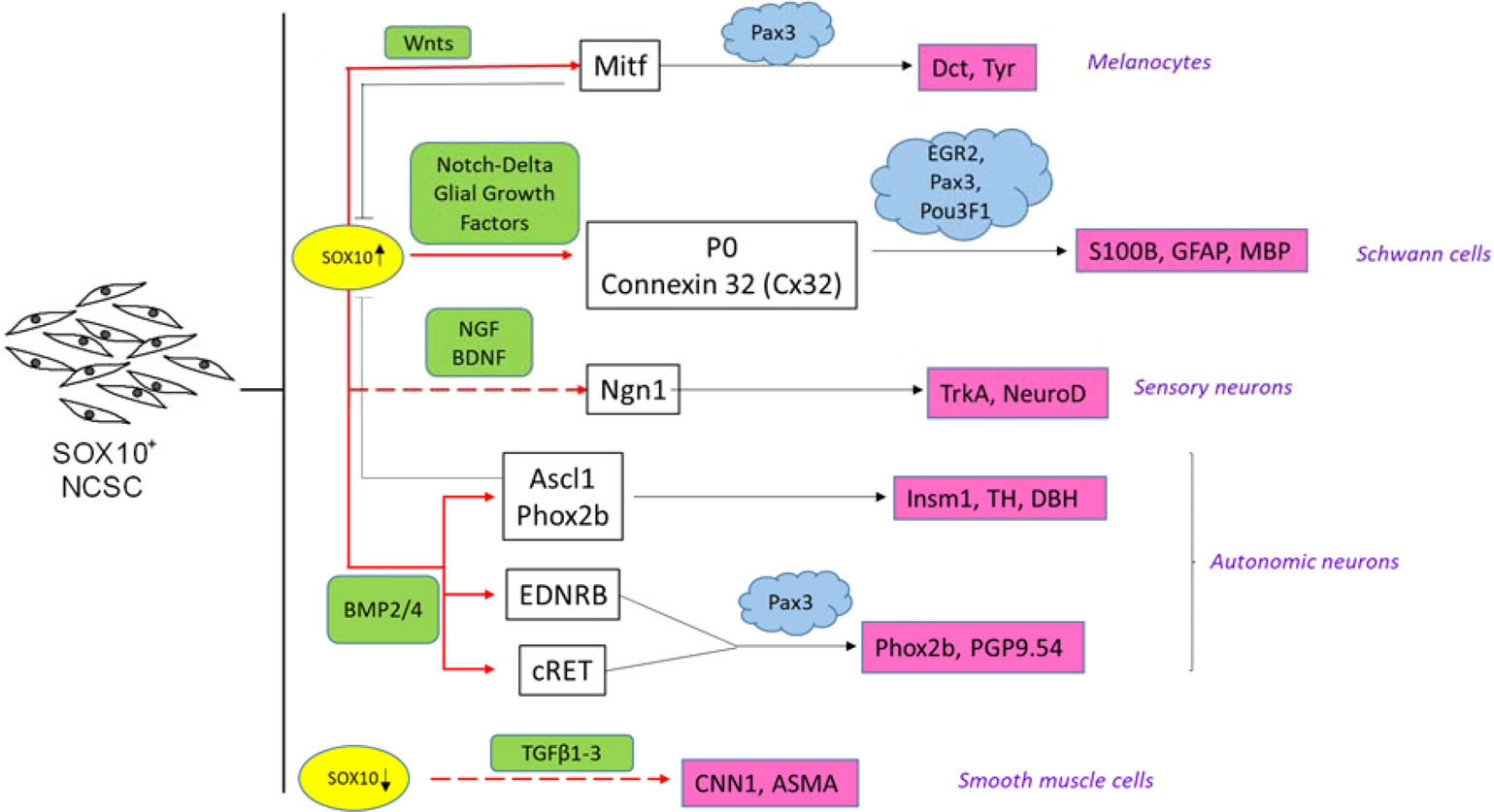
Role of Sox10 in differentiation to neural crest (NC)‐specific lineages. Sox10 is required for differentiation of NCs to neurons, melanocytes, and Schwann cells, but downregulated for smooth muscle cell differentiation. Red arrows represent direct binding sites of Sox10 to lineage specific transcription factors. Dashed red lines indicate no evidence for direct binding of Sox10. Green boxes represent extrinsic signals during differentiation. Factors that act synergistically with Sox10 are depicted in blue clouds. Downstream targets of gene regulation by Sox10 are depicted in pink boxes

Three main classes of signals, namely Wnts, BMP2/4, and TGFβ1/2/3, influence NC cell fate during and following migration (Figure 1).^73^, ^74^ Their interplay, timing, and relative intensity determine the proportion of various derivatives generated during development. Wnts and the downstream β‐catenin pathway have been found to promote pigment cell formation in zebrafish embryo, possibly through activation of *Nacre* (*Mitfa*), a gene necessary and sufficient for pigment cell formation; conversely, inhibition of Wnt signaling leads to generation of neurons at the expense of pigment cells.^75^, ^76^ Conditional deletion of β‐catenin in mice prevented sensory ganglion formation, whereas constitutive activation led to formation of sensory neurons at the expense of other NC derivatives.^77^, ^78^ The second class of signaling molecules, BMP2/4 are known to induce autonomic neurogenesis via expression of Ascl1 (MASH1), a basic helix‐loop‐helix transcription factor expressed in autonomic neuron precursors prior to differentiation. Interestingly, continuous BMP signaling is required for commitment of NCs to neuronal fate.^79^ Although BMP2/4 have been identified as factors promoting specification of NCs to an autonomic lineage, it does not prevent sensory neurogenesis in NCs prespecified to a sensory fate in vivo.^70^

The members of TGFβ superfamily—1/2/3 are also able to specify NC cell fates, through a different mechanism than BMP2/4, since they signal through a separate receptor complex and have different downstream effectors.^75^ TGFβ1/2/3 were shown to promote cardiac smooth muscle specification of rat NCs, as evidenced by the expression of smooth muscle actin.^74^ TGFβ1/2/3 are also expressed in the developing heart and thought to be active during the induction of cardiomyogenesis in NCs, as well as at final stages of cardiac cushion tissue formation.^80^

The development of SC from NCs results from exposure of NCs to neuregulin‐1 (NRG1, also known as Glial Growth Factor), an inductive signal of the neuregulin family. NRG1 has been found to suppress neuronal differentiation and specify NCs to a Schwann cell fate.^81^ The molecular mechanisms governing differentiation of NCs to SC are elaborated later in this review. The interplay of signaling pathways and their effect on different transcription factors are summarized in Figure 1.

## 4 MOLECULAR MECHANISMS UNDERLYING DIFFERENTIATION OF NC CELLS TO SC

During development, NCSC) delaminate from the neural tube and translocate to various regions of the embryo. Some localize adjacent to the developing nerves, where they differentiate into SCPs, which in turn give rise to a variety of peripheral glial cells that ultimately perform a plethora of functions linked to myelination, neuronal support, regulation of synaptic connectivity and sensory function. ErbB3/Nrg1 interactions and the transcription factor Zeb2 are involved in lineage specification and myelination^82^, ^83^, ^84^; however, the precise mechanisms dictating the fate acquisition from NC to SCP and mature myelinating or nonmyelinating SC, as well as the role of metabolic pathways in SC development and myelination, are not yet completely understood.

### 4.1 The role of Sox10, Pax3, and HDAC1 in NC lineage specification to SCP

The transcription factors Sox10 and Pax3 are responsible for maintenance of NC multipotency and for differentiation into SC and melanocytes. Although Sox10 is an important NC specifier,^85^ it is not the sole transcription factor setting the lineage specification and differentiation mechanisms in motion. Peripheral glial lineage specification is abolished when HDAC1/2 knockout mice (flanked by LoxP)^86^ are crossed with mice expressing Cre recombinase (Cre) under the control of Wnt1 promoter (Wnt1‐Cre).^87^, ^88^ Furthermore, HDAC1/2 has been shown to interact with Sox10 and activate two promoters,^89^, ^90^ the early lineage marker Myelin Protein Zero (MPZ or P0)^61^ and Pax3. In turn, a positive feedback loop is set in motion, where Pax3 and Sox10 activate the Sox10 MCS4 enhancer, to maintain high levels of Sox10 but also upregulate expression of Fabp7, another early SCP marker.^90^, ^91^ Interestingly, Sox10 expression precedes neuronal differentiation^92^, ^93^ but is downregulated during neurogenesis. In addition to Sox10 and Pax3, another key transcription factor FoxD3 plays a key role in glial fate acquisition, by biasing migrating NC against alternative melanocytic fate.^94^, ^95^, ^96^ Considering that Sox10 is also necessary for melanocytic differentiation and survival, the combined action of Sox10 and FoxD3 is necessary for commitment of NC to peripheral glial fate acquisition.

### 4.2 Path to myelination—Signaling during radial axonal sorting dictates lineage specification

Following HDAC1/2‐Sox10‐Pax3‐related signaling events, SCPs become immature SC, where the process of radial sorting takes place. Radial sorting refers to the process by which SC choose which axons to myelinate during development.^97^ It relies on the establishment of Schwann cell polarity and cytoskeletal remodeling through Schwann cell‐axon interactions, ultimately resulting in deposition of extracellular matrix (ECM), terminal Schwann cell differentiation, and myelin production. As expected, a variety of signals are necessary for these processes. ECM components like Laminin 211 and 411, Collagen XV and associated integrins α6β1, α7β1, as well as dystroglycan glycosylation enzymes play a pivotal role in attachment and sorting.^98^, ^99^, ^100^, ^101^, ^102^, ^103^, ^104^, ^105^ Intracellular signaling regulates cytoskeletal remodeling and radial sorting through molecules such as ILK, FAK, RhoGTPases, Rac1, Cdc42, Profilin, Merlin/NF2, and N‐WASp^106^, ^107^, ^108^, ^109^, ^110^, ^111^, ^112^, as summarized in Figure S2.

In addition, cell‐cell communications between neurons and SC regulate axonal myelination. Such interactions are mediated through Neuregulin 1 (Nrg1) type III on the surface of axons and ErbB2/3 on the surface of SC.^98^ Indeed inhibition of the ErbB pathway impaired axonal sorting in zebrafish nerves,^113^ strongly suggesting that ErbB2/3‐Nrg1 is necessary for Schwann cell radial sorting.^114^, ^115^, ^116^ Furthermore, the Erk/Akt signaling pathways downstream of Nrg1 were implicated in formation of abnormal Remak bundles containing unsorted large caliber axons, possibly through the inactivation of Gab1 (Grb2 associated binder 1), which in turn indirectly decreases Erk but not Akt phosphorylation.^117^ Finally, the Wnt/β‐catenin signaling has also been implicated in axonal radial sorting,^118^ as conditional inactivation of β‐catenin resulted in mild radial sorting defects and impaired lamellipodia formation.^119^

### 4.3 Metabolic signaling pathway networks in Schwann cell development and myelination

Recent studies revealed an increasingly important role for metabolism in neuronal and glial biology. Accumulating evidence supports the role of key metabolic pathways in SCP development^120^, ^121^; the importance of metabolic crosstalk between SC and axons^122^, ^123^, ^124^; and the contributions of metabolic abnormalities to the etiology of axonal degradation and myelin related disorders.^125^, ^126^, ^127^, ^128^, ^129^ Specifically, mTORC1, a multiprotein complex and major integrator of several growth factor (e.g., IGF‐1) signaling pathways such as PI3K/AKT and MAPK,^130^, ^131^ drives Schwann cell proliferation or myelin production, in a context‐specific manner that depends on developmental stage. On the one hand, mTORC1 impeded myelin production by promoting proliferation of immature SC and inhibiting their terminal differentiation and nerve development.^132^, ^133^, ^134^ On the other hand, myelin production was arrested in mTOR core kinase knockout mice and mTORC1 was identified as the cause of the deficiency due to its prime role in lipid formation and protein biosynthesis.^135^ In addition, LKB1, a serine threonine kinase upstream of AMPK, a key regulator of cellular energetics,^136^, ^137^, ^138^ plays a pivotal role in myelin production and axonal sorting in SC, with significant repercussions for PNS myelination. Collectively, these studies shed light on the etiology of myelopathies by providing links between metabolism and signaling during development, thereby providing valuable insights for development of therapeutic strategies that may involve the use of NC derived SC for cellular therapies.^139^, ^140^, ^141^

## 5 NCSCs: A CELL SOURCE FOR THE TREATMENT OF DEMYELINATING DISORDERS

Demyelinating disorders are generally defined as diseases resulting in loss of myelin from neuronal cells with relative preservation of axons. These disorders are a result of damage to myelin sheaths or to the cells that produce them. Demyelinated axons tend to degenerate, resulting in decrease or loss of neurological function. Demyelinating disorders affect both the CNS and PNS and can be caused by auto‐immunity, certain infectious agents, or genetic factors.^142^ Demyelinating diseases affecting the CNS include in multiple sclerosis (MS), acute‐disseminated encephalomyelitis, acute haemorrhagic leucoencephalitis, and progressive multifocal leukoencephalopathy, with MS being the most common disabling neurological illness affecting young and middle‐aged adults in North America and Europe.^143^ Demyelinating diseases affecting the PNS include the Guillain‐Barré syndrome, Charcot‐Marie‐Tooth disease, progressive inflammatory neuropathy, and copper deficiency. These diseases often result from loss of function of cells responsible for myelination—oligodendrocytes in the CNS or SC in the PNS. Although the causes and pathology of each of these diseases have been extensively studied, the likelihood of reoccurrence is high and treatment options are limited. Peripheral nerve injuries are also known to result in loss of myelin in and around the site of injury. The incidence of peripheral nerve injuries is fairly high, with a conservative estimates placing it between 13 and 23 per 100,000 people per year.^144^ Although nerve grafts have dominated the field of experimental treatment for peripheral nerve injuries, their use is often limited by technical difficulties, invasiveness, and mediocre outcomes.^145^

The last few decades have seen attempts to use SC expanded in culture for neural repair.^146^ SC play a central role in nerve repair—they become activated after a nerve injury and assume a primitive phenotype, upregulating genes encoding for the production of neurotropic factors and stimulating axonal regeneration. Additionally, once activated, SC produce ECM molecules such as collagen and laminin creating guided tunnels for axonal growth and regeneration.^145^ Various stem cell sources have been examined for the purpose of generating SC for the treatment of neurological diseases and peripheral nerve injury. Here, we provide a brief review of the therapeutic potential of these stem cells and discuss NCISC as an alternate cell source for Schwann cell engineering.

### 5.1 Stem cells for generation of autologous SC

Multipotent stem cells isolated from various adult sources may serve as an autologous source of SC. Multipotent mesenchymal stem cells (MSCs) have dominated this field, given their abundance and accessibility via minimally invasive procedures. Adipose tissue has been shown to be a rich source of MSCs that could be coaxed to generate SC in vitro and myelinate axons in the spinal cord in vivo.^147^, ^148^, ^149^, ^150^, ^151^, ^152^ However, these cells have limited in vitro expansion capacity, which hinders their clinical usefulness. Adult neural stem cells isolated from the brain have been shown to differentiate to S100/p75^NTR^ positive SC and improve axonal regeneration in mouse models of peripheral nerve injury.^153^, ^154^ However, neural tissue may not be an ideal source of stem cells due to its limited accessibility, invasive procurement, and frequent contamination with fibroblasts that may overpopulate the cultures upon cell expansion in vitro.^155^ Moreover, the potency and regenerative potential of neural stem cells may be affected by the neurodegenerative disease affecting the patient, calling the effectiveness of this cell source into question.

Since the discovery of the reprograming factors by Yamanaka et al,^156^ the focus of cell engineering has drastically shifted to reprogramming patient‐specific somatic cells to pluripotent stem cells). These iPSCs can then be coaxed to differentiate to any cell type by exposing them to signals that mimic embryonic development. In this regard, iPSCs have been considered as a potentially unlimited source of SC,^157^, ^158^, ^159^ which have been shown to induce axonal regrowth and facilitate myelination in mouse models of SCI.^160^, ^161^ ESCs derived from blastocyst stage embryos can also be used to produce SC, but significant ethical concerns are plaguing their clinical use. Clinical cases with patients undergoing cell‐based therapies for SCI have been evaluated in detail by Harrop et al.^162^ Though there has been considerable progress in the treatment of SCI and other neurodegenerative diseases, there clearly is a need of more accessible and easily expandable adult stem cell sources for derivation of myelinating SC.

### 5.2 Adult tissue–derived NCISCs for Schwann cell therapy

NC cells give rise to SC in vivo during development, thus making them one of the most suitable candidates as an autologous cell source of SC. ESCs and iPSCs go through an intermediate NC fate when coaxed to differentiate into SC, and some of these studies were discussed previously.^157^ Although most studies establish differentiation of pluripotent cells to mature SC, as evidenced by expression of S100β and MBP, few have demonstrated Schwann cell function in vivo.

As discussed above, several adult human tissues can also serve as potential sources of NCISCs. In one of the first studies, adult skin‐derived precursor cells (SKPs) were shown to have the potential to generate SC. SKPs are multipotent cells that are present in the skin dermis and express NC markers including Pax3, Snail, Slug, and NGFR.^28^ However, SKPs do not express the key NC transcription factors, SOX10 and FOXD3, and they have not been reported to give rise to melanocytes, chondrocytes, or osteocytes, all cells known to be derived from NCs. More recently, lineage tracing studies showed that murine SKPs are of mesodermal origin, in contrast to NCSC that originate from the ectoderm. Nevertheless, mesodermal SKPs gave rise to myelinating SC in the presence of signaling cues such as heregulin β, FGF, and forskolin, suggesting plasticity of developmentally defined lineage boundaries^163^ **(**Table 1 **)**. Furthermore, SKP‐derived SC were shown to myelinate axons as efficiently as CNS‐derived precursors, thus establishing them as a potent cell source for the treatment of SCI.^169^ Similarly, rat‐derived SKP‐SCs aided sensory,^171^ motor,^175^, ^176^ and behavioral recovery,^172^ and enhanced peripheral nerve regeneration compared with acellular nerve grafts.^174^, ^177^ Finally, SC generated from SKPs were used successfully for the treatment of incomplete cervical SCI.^170^, ^175^, ^178^

**Table 1:** Studies describing differentiation of adult‐tissue derived neural crest (NC) cells to Schwann cells (SC)

Source	Culture conditions	NC markers expressed	SC markers	Functionality	References
Human epidermal neural crest cells isolated from hair follicles	Alpha‐modified MEM containing retinoic acid (RA) (35 ng/mL), SB431542 (10 μM), rhFGF2 (10 ng/mL), PDGF‐BB (5 ng/mL), forskolin (5 μM) and neuregulin‐1 (200 ng/mL)	SOX10, p75	SOX10, KROX20, p75NTR, MBP and S100B	Coculture with primary dorsal root ganglion (DRG) neurons	Sakaue and Sieber‐Blum^164^
Human‐derived hair follicle cells	MesenPRO medium containing neuregulin‐1 (20 ng/mL), transfection with miR‐21 agonist agomir‐21	p75	S100β, GFAP		Ni et al^165^
Human neonatal foreskin	EBM2 basal medium, FBS (2%), ciliary neurotrophic factor (100 ng/mL), NRG1 (100 ng/mL), FGF2 (4 ng/mL), ascorbic acid (200 mg/mL), Glutamax (0.5X), SB431542 (10 mM)	SOX10, FOXD3, PAX3/7, SNAI2, TFAP2A, MSX1/2, cMYC, and SOX9	S100β, GFAP, MPZ, PLP1	NC migration toward DRG in chicken embryo	Bajpai et al^30^
Human‐derived dental pulp cells	αMEM supplemented with forskolin (5 μM), bFGF (10 ng/mL), PDGF (5 ng/mL), recombinant human neuregulin‐β1 (200 ng/mL)	CD271, SOX10, nestin	S100β		Al‐Zer et al^166^
Human bone marrow	DMEM/F12 supplemented with N2 supplement, CNTF (10 ng/mL), l bFGF (10 ng/m), dbcAMP (1 mM), neuregulin‐1β (20 ng/mL)	Nestin, Sox9, TWIST, SLUG, p75, SNAIL1, Brn3a, MSI1	S100β, MBP, P0	NC migration toward DRG in chicken embryo	Coste et al^167^
Human skin mesenchymal stem cells	Alpha‐MEM containing FBS (10%), RA (35 ng/mL), forskolin (5 μM), rh‐FGF‐2 (10 ng/mL), rhPDGF‐AA (5 ng/mL) and neuregulin‐1β (200 ng/mL)	p75NTR, Sox10, Notch1, integrin‐4α, Ap2α and Pax6	S100β, MBP	BDNF secretion by ELISA	Saulite et al.^168^
Postnatal rat bone marrow	DMEM/F12 supplemented with FBS (5%), all‐trans‐retinoic acid (tRA) (35 ng/mL), forskolin (5 mM), βheregulin‐1 (HRG‐1b) (200 ng/mL), FGF2 (10 ng/mL), PDGF‐AA (10 ng/mL), and N2 (1%)	p75, nestin	S100β, GFAP, CNPase p75	Coculture with primary dorsal root ganglion (DRG) neurons	Shi et al^11^
Human skin–derived precursors	Neurobasal medium containing 1% FBS plus 1% N2 supplement, 4 μM forskolin, and 10 ng/mL heregulin β	Pax3, snail, slug and NGFR	S100β, p75	—	Toma et al^28^
Mice skin–derived precursors	—	Nestin	MBP, GFAP	Myelination of spinal cord in mice	Mozafari et al^169^
Neonatal SKPs	—	—	—	Myelination of spinal cord in rats	Vasudeva et al^170^
Rodent/human skin mesenchymal precursors	DMEM/F12 at 3:1, N2 supplement (2%), neuregulin‐1β (10 ng/mL), and forskolin (4 μM)	—	GFAP, S100β, P0, p75NTR, and NECL4	Alignment of MBP expressing Schwann cells in vivo	Krause et al.^163^
Rat SKPs	—	—	p75	Sensory (thermal) recovery, axonal regeneration	Shakhbazau et al^171^
Rat SKPs	DMEM/F12, Neuregulin (50 ng/mL), forskolin (5 μM), N2 supplement (1%)	—	p75, nestin, MBP	Integration and ensheathment of regenerating axons and myelination, behavioral recovery	Khuong et al^172^
Mouse SKPs	Serum‐free DMEM/F12 supplemented with dibutyryl cyclic AMP (10 mM), bFGF (10 ng/mL), and neuregulin (20 ng/mL)	—	GFAP, S100β	—	Kanget al^173^
Rat SKPs	DMEM/F12 media with forksolin (4 μM), neuregulin‐1 (10 ng/mL), N2 supplement (1%)	—	S100β, p75	Secretion of bioactive neurotrophins, axonal regeneration	Walsh et al^174^
Rat SKPs	DMEM/F‐12 (3:1) containing forskolin (5 μM), neuregulin‐1 (50 ng/mL), N2 supplement (1%)	—	S100β, p75, P0	Axonal growth and myelination	Biernaskie et al^175^

Abbreviations: BDNF, brain‐derived neurotrophic factor; DMEM, Dulbecco's Modified Eagle Medium; ELISA, enzyme‐linked immunosorbent assay; FGF, fibroblast growth factor; GFAP, glial fibrillary acidic protein; MBP, myelin basic protein; NRG1, neuregulin‐1; SKP, skin‐derived precursor cell.

Recently, NCISCs were isolated from the bulge of hair follicles and coaxed to differentiate into SC, a process that required NRG1 and micro‐RNA miR‐21.^165^ These SC expressed SOX10, p75NTR (NGFR), KROX20, MBP, and S100β and interacted with axons in a DRG coculture model, thus providing evidence of their functionality. Dental pulp stem cells derived from human wisdom teeth have also been shown to resemble NCSC and were capable of differentiating into S100β‐expressing SC.^166^ Finally, human and rat bone marrow–derived NC‐like cells were also capable of differentiating into SC in vitro.^11^, ^167^

Recently, our laboratory showed that cultures of KC from interfollicular epidermis could be the source of NClSCs (termed KC‐NC), after induction with specific chemical cues.^30^ KC‐derived NCISCs could be coaxed to differentiate into functional neurons, SC, melanocytes, osteocytes, chondrocytes, adipocytes, and smooth muscle cells in vitro. Most notably, upon transplantation into chick embryos, KC‐NCs migrated along stereotypical pathways and gave rise to multiple NC derivatives, providing strong support of their NC‐like phenotype. Specifically, these cells gave rise to BLBP+ glial cells in ovo and were localized around the axon bundles. Interestingly, KC‐NC from aged donors maintained the same differentiation potential in vitro and in ovo,^32^ indicating the potential of adult epidermis as a source of KC‐NC for the treatment of neurodegenerative diseases. Current studies in our laboratory employ the hypomyelinating *Shiverer/Rag2−/−* mouse—a model of congenital hypomyelinating disease that has become the gold standard for the assessment of myelinating cell preparations—to examine whether KC‐NC or SC derived from them can be used to myelinate axons and rescue the *shiverer* phenotype. Table 1 depicts studies describing differentiation of adult tissue–derived NClSCs to SC.

## 6 CONCLUSION AND FUTURE PERSPECTIVES

NC cells have attracted great interest due to their ability to differentiate into multiple cell types.^156^ Although NC stem cells are a transient cell population in developing embryos, multiple investigators have isolated cells with NC‐like characteristics, including transcriptional profile and differentiation potential, from a variety of adult tissues like DRG, bone marrow, skin, carotid body, whisker pad, heart, gut, and cranial tissues like cornea, iris, hard palate, dental pulp, and oral mucosa.^1^ Regardless of source, NC‐like cells have been coaxed to differentiate into SC, neurons, chondrocytes, smooth muscle cells, and even cardiomyocytes,^2^, ^179^, ^180^ providing a multipotent stem cell source for the treatment of demyelinating and other neurodegenerative disorders. Patient‐derived NCISCs harboring mutations for neurogenic diseases may also be used to study disease pathogenesis as well as provide a platform for drug screening further increasing their clinical potential.^1^

The use of SC derived from adult tissue–derived NClSCs for the treatment of SCI has gained momentum in recent years, after demonstration of successful myelination of axons in vitro and in vivo. Though these results are very promising, most studies use cells derived from mice or rats, which are implanted in a mouse contusion model of SCI. However, the potential of these cells for the treatment of demyelinating disease in large animal models or humans has yet to be established. Furthermore, most studies have focused on myelination after SCI and not treatment of demyelinating disorders of the CNS or PNS, leaving the field fairly unexplored in this domain. Finally, isolation of NCISCs from patients suffering from neurodegenerative disorders and their differentiation to SC and neurons may help identify abnormalities in myelin production and nerve conduction, aiding in better disease diagnosis and development of patient‐specific therapies.

## CONFLICT OF INTEREST

The authors indicated no potential conflicts of interest.

## AUTHOR CONTRIBUTIONS

P.M. wrote the manuscript, specifically parts on introduction to the topic, sources of neural crest and their genetic regulation, their application for treatment of demyelinating disorders and made the manuscript figures. G.T. wrote the manuscript, specifically mechanisms underlying differentiation of neural crest cells to Schwann cells and made the manuscript figures. M.E.B. wrote the manuscript, specifically content on how Schwann cell precursors contribute to neural crest derivatives, and gene regulatory networks controlling lineage diversification. S.T.A. conceived overall review content, writing, and editing the manuscript and project supervision.

## Supporting information


**Figure S1:** Adult sources for in vitro expansion of multipotent stem cells exhibiting an NC‐like phenotype. From the above mentioned sources, only cells from dental pulp and hair follicles have been successfully isolated and studied in humans
**Figure S2:** A Neural Crest Stem Cell's journey to myelination. Delaminating, post migratory NC follow Wnt and FGF signals and upregulate key transcription factors like Sox10, FoxD3 and Pax3. By recruiting HDAC1/2, myelin protein 0 is upregulated leading to a Schwann cell precursor phenotype. During this immature Schwann cell stage, Schwann cells initially approach multiple axons and after undergoing significant cytoskeletal reorganization, each one myelinates a single axon, a process referred to as axonal sorting. Finally, the mature Schwann cell envelopes along the neuronal axon with myelin sheath to facilitate saltatory electrical impulse conduction.

## Data Availability

The data that support the findings of this study are available from the corresponding author upon reasonable request.

## References

[1] Liu JA , Cheung M . Neural crest stem cells and their potential therapeutic applications. Dev Biol. 2016;419:199‐216. 27640086 10.1016/j.ydbio.2016.09.006

[2] Martik ML , Bronner ME . Regulatory logic underlying diversification of the neural crest. Trends Genet. 2017;33:715‐727. 28851604 10.1016/j.tig.2017.07.015 PMC5610108

[3] O'Rahilly R , Müller F . The development of the neural crest in the human. J Anat. 2007;211:335‐351. 17848161 10.1111/j.1469-7580.2007.00773.x PMC2375817

[4] Stemple DL , Anderson DJ . Isolation of a stem cell for neurons and glia from the mammalian neural crest. Cell. 1992;71:973‐985. 1458542 10.1016/0092-8674(92)90393-q

[5] Betancur P , Bronner‐Fraser M , Sauka‐Spengler T . Assembling neural crest regulatory circuits into a gene regulatory network. Annu Rev Cell Dev Biol. 2010;26:581‐603. 19575671 10.1146/annurev.cellbio.042308.113245 PMC4040144

[6] Sauka‐Spengler T , Bronner‐Fraser M . A gene regulatory network orchestrates neural crest formation. Nat Rev Mol Cell Biol. 2008;9:557‐568. 18523435 10.1038/nrm2428

[7] Thiery JP , Sleeman JP . Complex networks orchestrate epithelial–mesenchymal transitions. Nat Rev Mol Cell Biol. 2006;7:131‐142. 16493418 10.1038/nrm1835

[8] Le Douarin N , Kalcheim C . The Neural Crest. Cambridge, UK: Cambridge University Press; 1999.

[9] Morrison SJ , White PM , Zock C , Anderson DJ . Prospective identification, isolation by flow cytometry, and in vivo self‐renewal of multipotent mammalian neural crest stem cells. Cell. 1999;96:737‐749. 10089888 10.1016/s0092-8674(00)80583-8

[10] Nagoshi N , Shibata S , Kubota Y , et al. Ontogeny and multipotency of neural crest‐derived stem cells in mouse bone marrow, dorsal root ganglia, and whisker pad. Cell Stem Cell. 2008;2:392‐403. 18397758 10.1016/j.stem.2008.03.005

[11] Shi H , Gong Y , Qiang L , et al. Derivation of Schwann cell precursors from neural crest cells resident in bone marrow for cell therapy to improve peripheral nerve regeneration. Biomaterials. 2016;89:25‐37. 26946403 10.1016/j.biomaterials.2016.02.029

[12] Hassell JR , Birk DE . The molecular basis of corneal transparency. Exp Eye Res. 2010;91:326‐335. 20599432 10.1016/j.exer.2010.06.021 PMC3726544

[13] Gage PJ , Rhoades W , Prucka SK , Hjalt T . Fate maps of neural crest and mesoderm in the mammalian eye. Invest Ophthalmol Vis Sci. 2005;46:4200‐4208. 16249499 10.1167/iovs.05-0691

[14] Whikehart, D. , Corneal Endothelium: Overview. 2010.

[15] Williams AL , Bohnsack BL . Neural crest derivatives in ocular development: discerning the eye of the storm. Birth Defects Res C Embryo Today. 2015;105:87‐95. 26043871 10.1002/bdrc.21095 PMC5262495

[16] Lwigale PY , Cressy PA , Bronner‐Fraser M . Corneal keratocytes retain neural crest progenitor cell properties. Dev Biol. 2005;288:284‐293. 16263107 10.1016/j.ydbio.2005.09.046

[17] Du Y , et al. Multipotent stem cells in human corneal stroma. Stem Cells. 2005;23:1266‐1275. 16051989 10.1634/stemcells.2004-0256 PMC1941788

[18] Joseph NM , He S , Quintana E , Kim YG , Núñez G , Morrison SJ . Enteric glia are multipotent in culture but primarily form glia in the adult rodent gut. J Clin Invest. 2011;121:3398‐3411. 21865643 10.1172/JCI58186 PMC3163971

[19] Kruger GM , Mosher JT , Bixby S , Joseph N , Iwashita T , Morrison SJ . Neural crest stem cells persist in the adult gut but undergo changes in self‐renewal, neuronal subtype potential, and factor responsiveness. Neuron. 2002;35:657‐669. 12194866 10.1016/s0896-6273(02)00827-9 PMC2728576

[20] Dettmann HM , Zhang Y , Wronna N , et al. Isolation, expansion and transplantation of postnatal murine progenitor cells of the enteric nervous system. PLoS One. 2014;9:e97792. 10.1371/journal.pone.0097792 PMC4037209 24871092

[21] Hotta R , Stamp LA , Foong JPP , et al. Transplanted progenitors generate functional enteric neurons in the postnatal colon. J Clin Invest. 2013;123:1182‐1191. 23454768 10.1172/JCI65963 PMC3582137

[22] Ito K , Sieber‐Blum M . In vitro clonal analysis of quail cardiac neural crest development. Dev Biol. 1991;148:95‐106. 1936578 10.1016/0012-1606(91)90320-3

[23] Tomita Y , Matsumura K , Wakamatsu Y , et al. Cardiac neural crest cells contribute to the dormant multipotent stem cell in the mammalian heart. J Cell Biol. 2005;170:1135‐1146. 16186259 10.1083/jcb.200504061 PMC2171522

[24] El‐Helou V , et al. Cardiac resident nestin+ cells participate in reparative vascularisation. J Cell Physiol. 2013;228:1844‐1853. 23459851 10.1002/jcp.24345

[25] Fernandes KJ , et al. A dermal niche for multipotent adult skin‐derived precursor cells. Nat Cell Biol. 2004;6:1082‐1093. 15517002 10.1038/ncb1181

[26] Sieber‐Blum M , Grim M , Hu YF , Szeder V . Pluripotent neural crest stem cells in the adult hair follicle. Dev Dyn. 2004;231:258‐269. 15366003 10.1002/dvdy.20129

[27] Toma JG , Akhavan M , Fernandes KJL , et al. Isolation of multipotent adult stem cells from the dermis of mammalian skin. Nat Cell Biol. 2001;3:778‐784. 11533656 10.1038/ncb0901-778

[28] Toma JG , McKenzie IA , Bagli D , Miller FD . Isolation and characterization of multipotent skin‐derived precursors from human skin. Stem Cells. 2005;23:727‐737. 15917469 10.1634/stemcells.2004-0134

[29] Sieber‐Blum M , Schnell L , Grim M , Hu YF , Schneider R , Schwab ME . Characterization of epidermal neural crest stem cell (EPI‐NCSC) grafts in the lesioned spinal cord. Mol Cell Neurosci. 2006;32:67‐81. 16626970 10.1016/j.mcn.2006.02.003

[30] Bajpai VK , Kerosuo L , Tseropoulos G , et al. Reprogramming postnatal human epidermal keratinocytes toward functional neural crest fates. Stem Cells. 2017;35:1402‐1415. 28142205 10.1002/stem.2583 PMC5543412

[31] Tseropoulos G , Moghadasi Boroujeni S , Bajpai VK , Lei P , Andreadis ST . Derivation of neural crest stem cells from human epidermal keratinocytes requires FGF‐2, IGF‐1, and inhibition of TGF‐β1. Bioeng Transl Med. 2018;3:256‐264. 30377664 10.1002/btm2.10109 PMC6195909

[32] Boroujeni SM , et al. Neural crest stem cells from human epidermis of aged donors maintain their multipotency in vitro and in vivo. Sci Rep. 2019;9:9750. 31278326 10.1038/s41598-019-46140-9 PMC6611768

[33] Adameyko I , Lallemend F , Aquino JB , et al. Schwann cell precursors from nerve innervation are a cellular origin of melanocytes in skin. Cell. 2009;139:366‐379. 19837037 10.1016/j.cell.2009.07.049

[34] Dyachuk V , Furlan A , Shahidi MK , et al. Parasympathetic neurons originate from nerve‐associated peripheral glial progenitors. Science. 2014;345:82‐87. 24925909 10.1126/science.1253281

[35] Espinosa‐Medina I , Jevans B , Boismoreau F , et al. Dual origin of enteric neurons in vagal Schwann cell precursors and the sympathetic neural crest. Proc Natl Acad Sci. 2017;114:11980‐11985. 29078343 10.1073/pnas.1710308114 PMC5692562

[36] Uesaka T , Nagashimada M , Enomoto H . Neuronal differentiation in Schwann cell lineage underlies postnatal neurogenesis in the enteric nervous system. J Neurosci. 2015;35:9879‐9888. 26156989 10.1523/JNEUROSCI.1239-15.2015 PMC6605410

[37] Jaegle M , Mandemakers W , Broos L , et al. The POU factor Oct‐6 and Schwann cell differentiation. Science. 1996;273:507‐510. 8662541 10.1126/science.273.5274.507

[38] Buchstaller J , Sommer L , Bodmer M , Hoffmann R , Suter U , Mantei N . Efficient isolation and gene expression profiling of small numbers of neural crest stem cells and developing Schwann cells. J Neurosci. 2004;24:2357‐2365. 15014110 10.1523/JNEUROSCI.4083-03.2004 PMC6729482

[39] Takahashi M , Osumi N . Identification of a novel type II classical cadherin: rat cadherin19 is expressed in the cranial ganglia and Schwann cell precursors during development. Dev Dyn. 2005;232:200‐208. 15580626 10.1002/dvdy.20209

[40] D'antonio M , et al. Gene profiling and bioinformatic analysis of Schwann cell embryonic development and myelination. Glia. 2006;53:501‐515. 16369933 10.1002/glia.20309

[41] Li J , Habbes HW , Eiberger J , Willecke K , Dermietzel R , Meier C . Analysis of connexin expression during mouse Schwann cell development identifies connexin29 as a novel marker for the transition of neural crest to precursor cells. Glia. 2007;55:93‐103. 17024657 10.1002/glia.20427

[42] Furlan A , et al. Multipotent peripheral glial cells generate neuroendocrine cells of the adrenal medulla. Science. 2017;357:eaal3753. 10.1126/science.aal3753 PMC6013038 28684471

[43] Jessen KR , Mirsky R . Schwann cell precursors; multipotent glial cells in embryonic nerves. Front Mol Neurosci. 2019;12:69. 30971890 10.3389/fnmol.2019.00069 PMC6443887

[44] Rada‐Iglesias A , Bajpai R , Prescott S , Brugmann SA , Swigut T , Wysocka J . Epigenomic annotation of enhancers predicts transcriptional regulators of human neural crest. Cell Stem Cell. 2012;11:633‐648. 22981823 10.1016/j.stem.2012.07.006 PMC3751405

[45] Soldatov R , et al. Spatiotemporal structure of cell fate decisions in murine neural crest. Science. 2019;364(6444):eaas9536. 10.1126/science.aas9536 31171666

[46] Meulemans D , Bronner‐Fraser M . Gene‐regulatory interactions in neural crest evolution and development. Dev Cell. 2004;7:291‐299. 15363405 10.1016/j.devcel.2004.08.007

[47] Simões‐Costa M , Bronner ME . Establishing neural crest identity: a gene regulatory recipe. Development. 2015;142:242‐257. 25564621 10.1242/dev.105445 PMC4302844

[48] Stewart RA , Arduini BL , Berghmans S , et al. Zebrafish foxd3 is selectively required for neural crest specification, migration and survival. Dev Biol. 2006;292:174‐188. 16499899 10.1016/j.ydbio.2005.12.035

[49] Kelsh RN . Sorting out Sox10 functions in neural crest development. Bioessays. 2006;28:788‐798. 16927299 10.1002/bies.20445

[50] Sauka‐Spengler T , Bronner‐Fraser M . Insights from a sea lamprey into the evolution of neural crest gene regulatory network. Biol Bull. 2008;214:303‐314. 18574106 10.2307/25470671

[51] Kim J , Lo L , Dormand E , Anderson DJ . SOX10 maintains multipotency and inhibits neuronal differentiation of neural crest stem cells. Neuron. 2003;38:17‐31. 12691661 10.1016/s0896-6273(03)00163-6

[52] Carney TJ , Dutton KA , Greenhill E , et al. A direct role for Sox10 in specification of neural crest‐derived sensory neurons. Development. 2006;133:4619‐4630. 17065232 10.1242/dev.02668

[53] Zhu L , Lee HO , Jordan CRS , Cantrell VA , Southard‐Smith EM , Shin MK . Spatiotemporal regulation of endothelin receptor‐B by SOX10 in neural crest–derived enteric neuron precursors. Nat Genet. 2004;36:732‐737. 15170213 10.1038/ng1371

[54] Schreiner S , Cossais F , Fischer K , et al. Hypomorphic Sox10 alleles reveal novel protein functions and unravel developmental differences in glial lineages. Development. 2007;134:3271‐3281. 17699610 10.1242/dev.003350

[55] Svaren J , Meijer D . The molecular machinery of myelin gene transcription in Schwann cells. Glia. 2008;56:1541‐1551. 18803322 10.1002/glia.20767 PMC2930200

[56] Finzsch M , Schreiner S , Kichko T , et al. Sox10 is required for Schwann cell identity and progression beyond the immature Schwann cell stage. J Cell Biol. 2010;189:701‐712. 20457761 10.1083/jcb.200912142 PMC2872908

[57] Peirano RI , Goerich DE , Riethmacher D , Wegner M . Protein zero gene expression is regulated by the glial transcription factor Sox10. Mol Cell Biol. 2000;20:3198‐3209. 10757804 10.1128/mcb.20.9.3198-3209.2000 PMC85614

[58] Bondurand N , Girard M , Pingault V , Lemort N , Dubourg O , Goossens M . Human Connexin 32, a gap junction protein altered in the X‐linked form of Charcot–Marie–Tooth disease, is directly regulated by the transcription factor SOX10. Hum Mol Genet. 2001;10:2783‐2795. 11734543 10.1093/hmg/10.24.2783

[59] Weston JA . 6 sequential segregation and fate of developmentally restricted intermediate cell populations in the neural crest lineage. Curr Top Dev Biol. 1991;25:133‐153. 1660392 10.1016/s0070-2153(08)60414-7

[60] Wehrle‐Haller B , Weston JA . Receptor tyrosine kinase‐dependent neural crest migration in response to differentially localized growth factors. Bioessays. 1997;19:337‐345. 9136631 10.1002/bies.950190411

[61] Hagedorn L , Suter U , Sommer L . P0 and PMP22 mark a multipotent neural crest‐derived cell type that displays community effects in response to TGF‐beta family factors. Development. 1999;126:3781‐3794. 10433908 10.1242/dev.126.17.3781

[62] Sieber‐Blum M , Ito K , Richardson MK , Langtimm CJ , Duff RS . Distribution of pluripotent neural crest cells in the embryo and the role of brain‐derived neurotrophic factor in the commitment to the primary sensory neuron lineage. Dev Neurobiol. 1993;24:173‐184. 10.1002/neu.480240205 8445386

[63] Le Douarin NM , Dupin E . Cell lineage analysis in neural crest ontogeny. Dev Neurobiol. 1993;24:146‐161. 10.1002/neu.480240203 8445384

[64] Beall AC , Rosenquist TH . Smooth muscle cells of neural crest origin form the aorticopulmonary septum in the avian embryo. Anat Rec. 1990;226:360‐366. 2327605 10.1002/ar.1092260313

[65] Phillips MT , Kirby ML , Forbes G . Analysis of cranial neural crest distribution in the developing heart using quail‐chick chimeras. Circ Res. 1987;60:27‐30. 3568286 10.1161/01.res.60.1.27

[66] Fantauzzo KA , Soriano P . Receptor tyrosine kinase signaling: regulating neural crest development one phosphate at a time. Curr Top Dev Biol. 2015;111:135‐182. 25662260 10.1016/bs.ctdb.2014.11.005 PMC4363133

[67] Henion PD , Garner AS , Large TH , Weston JA . trkC‐mediated NT‐3 signaling is required for the early development of a subpopulation of neurogenic neural crest cells. Dev Biol. 1995;172:602‐613. 8612975 10.1006/dbio.1995.8054

[68] Meyer D , Birchmeier C . Multiple essential functions of neuregulin in development. Nature. 1995;378:386‐390. 7477375 10.1038/378386a0

[69] Schatteman GC , Morrison‐Graham K , van Koppen A , Weston JA , Bowen‐Pope DF . Regulation and role of PDGF receptor alpha‐subunit expression during embryogenesis. Development. 1992;115:123‐131. 1322269 10.1242/dev.115.1.123

[70] Greenwood AL , Turner EE , Anderson DJ . Identification of dividing, determined sensory neuron precursors in the mammalian neural crest. Development. 1999;126:3545‐3559. 10409501 10.1242/dev.126.16.3545

[71] Ma Q , Fode C , Guillemot F , Anderson DJ . Neurogenin1 and neurogenin2 control two distinct waves of neurogenesis in developing dorsal root ganglia. Genes Dev. 1999;13:1717‐1728. 10398684 10.1101/gad.13.13.1717 PMC316844

[72] Reedy MV , Faraco CD , Erickson CA . The delayed entry of thoracic neural crest cells into the dorsolateral path is a consequence of the late emigration of melanogenic neural crest cells from the neural tube. Dev Biol. 1998;200:234‐246. 9705230 10.1006/dbio.1998.8963

[73] LaBonne C , Bronner‐Fraser M . Neural crest induction in Xenopus: evidence for a two‐signal model. Development. 1998;125:2403‐2414. 9609823 10.1242/dev.125.13.2403

[74] Shah NM , Groves AK , Anderson DJ . Alternative neural crest cell fates are instructively promoted by TGFβ superfamily members. Cell. 1996;85:331‐343. 8616889 10.1016/s0092-8674(00)81112-5

[75] Dorsky RI , Moon RT , Raible DW . Environmental signals and cell fate specification in premigratory neural crest. Bioessays. 2000;22:708‐716. 10918301 10.1002/1521-1878(200008)22:8<708::AID-BIES4>3.0.CO;2-N

[76] Dorsky RI , Moon RT , Raible DW . Control of neural crest cell fate by the Wnt signalling pathway. Nature. 1998;396:370‐373. 9845073 10.1038/24620

[77] Hari L , Brault V , Kléber M , et al. Lineage‐specific requirements of β‐catenin in neural crest development. J Cell Biol. 2002;159:867‐880. 12473692 10.1083/jcb.200209039 PMC2173383

[78] Lee H‐Y , Kléber M , Hari L , et al. Instructive role of Wnt/ß‐catenin in sensory fate specification in neural crest stem cells. Science. 2004;303:1020‐1023. 14716020 10.1126/science.1091611

[79] Lo L , Sommer L , Anderson DJ . MASH1 maintains competence for BMP2‐induced neuronal differentiation in post‐migratory neural crest cells. Curr Biol. 1997;7:440‐450. 9197246 10.1016/s0960-9822(06)00191-6

[80] Dickson MC , Slager HG , Duffie E , Mummery CL , Akhurst RJ . RNA and protein localisations of TGF beta 2 in the early mouse embryo suggest an involvement in cardiac development. Development. 1993;117:625‐639. 7687212 10.1242/dev.117.2.625

[81] Shah NM , Marchionni MA , Isaacs I , Stroobant P , Anderson DJ . Glial growth factor restricts mammalian neural crest stem cells to a glial fate. Cell. 1994;77:349‐360. 7910115 10.1016/0092-8674(94)90150-3

[82] Aquino JB , Sierra R . Schwann cell precursors in health and disease. Glia. 2018;66:465‐476. 29124786 10.1002/glia.23262

[83] Luca Franco Castelnovo VB , Melfi S , Ballabio M , Colleoni D , Magnaghi V . Schwann cell development, maturation and regeneration: a focus on classic and emerging intracellular signaling pathways. Neural Regen Res. 2017;12:1013‐1023. 28852375 10.4103/1673-5374.211172 PMC5558472

[84] Bronner ME , Simões‐Costa M . The neural crest migrating into the twenty‐first century. Curr Top Dev Biol. 2016;116:115‐134. 26970616 10.1016/bs.ctdb.2015.12.003 PMC5100668

[85] Weider M , Wegner M . SoxE factors: transcriptional regulators of neural differentiation and nervous system development. Semin Cell Dev Biol. 2017;63:35‐42. 27552919 10.1016/j.semcdb.2016.08.013

[86] Jacob C , Lotscher P , Engler S , et al. HDAC1 and HDAC2 control the specification of neural crest cells into peripheral glia. J Neurosci. 2014;34:6112‐6122. 24760871 10.1523/JNEUROSCI.5212-13.2014 PMC3996228

[87] Auerbach R . Analysis of the developmental effects of a lethal mutation in the house mouse. J Exp Zool. 1954;127:305‐329.

[88] Franz T . Defective ensheathment of motoric nerves in the Splotch mutant mouse. Cells Tissues Organs. 1990;138:246‐253. 10.1159/000146947 2389670

[89] Antonellis A , Huynh JL , Lee‐Lin SQ , et al. Identification of neural crest and glial enhancers at the mouse Sox10 locus through transgenesis in zebrafish. PLoS Genet. 2008;4:e1000174. 10.1371/journal.pgen.1000174 PMC2518861 18773071

[90] Wahlbuhl M , Reiprich S , Vogl MR , Bösl MR , Wegner M . Transcription factor Sox10 orchestrates activity of a neural crest‐specific enhancer in the vicinity of its gene. Nucleic Acids Res. 2011;40:88‐101. 21908409 10.1093/nar/gkr734 PMC3245941

[91] Kurtz A , Zimmer A , Schnütgen F , Brüning G , Spener F , Müller T . The expression pattern of a novel gene encoding brain‐fatty acid binding protein correlates with neuronal and glial cell development. Development. 1994;120:2637‐2649. 7956838 10.1242/dev.120.9.2637

[92] Britsch S , Goerich DE , Riethmacher D , et al. The transcription factor Sox10 is a key regulator of peripheral glial development. Genes Dev. 2001;15:66‐78. 11156606 10.1101/gad.186601 PMC312607

[93] Kuhlbrodt K , Herbarth B , Sock E , Hermans‐Borgmeyer I , Wegner M . Sox10, a novel transcriptional modulator in glial cells. J Neurosci. 1998;18:237‐250. 9412504 10.1523/JNEUROSCI.18-01-00237.1998 PMC6793382

[94] Nitzan E , Krispin S , Pfaltzgraff ER , Klar A , Labosky PA , Kalcheim C . A dynamic code of dorsal neural tube genes regulates the segregation between neurogenic and melanogenic neural crest cells. Development. 2013;140:2269‐2279. 23615280 10.1242/dev.093294 PMC3653553

[95] Nitzan E , Pfaltzgraff ER , Labosky PA , Kalcheim C . Neural crest and Schwann cell progenitor‐derived melanocytes are two spatially segregated populations similarly regulated by Foxd3. Proc Natl Acad Sci USA. 2013;110:12709‐12714. 23858437 10.1073/pnas.1306287110 PMC3732929

[96] Thomas AJ , Erickson CA . FOXD3 regulates the lineage switch between neural crest‐derived glial cells and pigment cells by repressing MITF through a non‐canonical mechanism. Development. 2009;136:1849‐1858. 19403660 10.1242/dev.031989 PMC2680109

[97] Feltri ML , Poitelon Y , Previtali SC . How Schwann cells sort axons: new concepts. Neuroscientist. 2016;22:252‐265. 25686621 10.1177/1073858415572361 PMC5181106

[98] Berti C , Bartesaghi L , Ghidinelli M , et al. Non‐redundant function of dystroglycan and β1 integrins in radial sorting of axons. Development. 2011;138:4025‐4037. 21862561 10.1242/dev.065490 PMC3160097

[99] Feltri ML , Porta DG , Previtali SC , et al. Conditional disruption of β1 integrin in Schwann cells impedes interactions with axons. J Cell Biol. 2002;156:199‐210. 11777940 10.1083/jcb.200109021 PMC2173589

[100] Occhi S , Zambroni D , del Carro U , et al. Both laminin and Schwann cell dystroglycan are necessary for proper clustering of sodium channels at nodes of Ranvier. J Neurosci. 2005;25:9418‐9427. 16221851 10.1523/JNEUROSCI.2068-05.2005 PMC1409814

[101] Patton BL , Wang B , Tarumi YS , Seburn KL , Burgess RW . A single point mutation in the LN domain of LAMA2 causes muscular dystrophy and peripheral amyelination. J Cell Sci. 2008;121:1593‐1604. 18430779 10.1242/jcs.015354

[102] Pellegatta M , et al. α6β1 and α7β1 integrins are required in Schwann cells to sort axons. J Neurosci. 2013;33:17995‐18007. 24227711 10.1523/JNEUROSCI.3179-13.2013 PMC3893357

[103] Saito F , Masaki T , Saito Y , et al. Defective peripheral nerve myelination and neuromuscular junction formation in fukutin‐deficient chimeric mice. J Neurochem. 2007;101:1712‐1722. 17326765 10.1111/j.1471-4159.2007.04462.x

[104] Wallquist W , Plantman S , Thams S , et al. Impeded interaction between Schwann cells and axons in the absence of laminin α4. J Neurosci. 2005;25:3692‐3700. 15814800 10.1523/JNEUROSCI.5225-04.2005 PMC6725372

[105] Yang D , Bierman J , Tarumi YS , et al. Coordinate control of axon defasciculation and myelination by laminin‐2 and‐8. J Cell Biol. 2005;168:655‐666. 15699217 10.1083/jcb.200411158 PMC2171752

[106] Benninger Y , Thurnherr T , Pereira JA , et al. Essential and distinct roles for cdc42 and rac1 in the regulation of Schwann cell biology during peripheral nervous system development. J Cell Biol. 2007;177:1051‐1061. 17576798 10.1083/jcb.200610108 PMC2064365

[107] Grove M , Komiyama NH , Nave KA , Grant SG , Sherman DL , Brophy PJ . FAK is required for axonal sorting by Schwann cells. J Cell Biol. 2007;176:277‐282. 17242067 10.1083/jcb.200609021 PMC2063954

[108] Guo L , Moon C , Niehaus K , Zheng Y , Ratner N . Rac1 controls Schwann cell myelination through cAMP and NF2/merlin. J Neurosci. 2012;32:17251‐17261. 23197717 10.1523/JNEUROSCI.2461-12.2012 PMC3601465

[109] Nodari A , Zambroni D , Quattrini A , et al. β1 integrin activates Rac1 in Schwann cells to generate radial lamellae during axonal sorting and myelination. J Cell Biol. 2007;177:1063‐1075. 17576799 10.1083/jcb.200610014 PMC2064366

[110] Novak N , Bar V , Sabanay H , et al. N‐WASP is required for membrane wrapping and myelination by Schwann cells. J Cell Biol. 2011;192:243‐250. 21263026 10.1083/jcb.201010013 PMC3172181

[111] Pereira JA , Benninger Y , Baumann R , et al. Integrin‐linked kinase is required for radial sorting of axons and Schwann cell remyelination in the peripheral nervous system. J Cell Biol. 2009;185:147‐161. 19349584 10.1083/jcb.200809008 PMC2700520

[112] Jin F , Dong B , Georgiou J , et al. N‐WASp is required for Schwann cell cytoskeletal dynamics, normal myelin gene expression and peripheral nerve myelination. Development. 2011;138:1329‐1337. 21385763 10.1242/dev.058677 PMC3188810

[113] Raphael AR , Lyons DA , Talbot WS . ErbB signaling has a role in radial sorting independent of Schwann cell number. Glia. 2011;59:1047‐1055. 21491500 10.1002/glia.21175 PMC3094506

[114] Nave K‐A , Salzer JL . Axonal regulation of myelination by neuregulin 1. Curr Opin Neurobiol. 2006;16:492‐500. 16962312 10.1016/j.conb.2006.08.008

[115] Newbern J , Birchmeier C . Nrg1/ErbB signaling networks in Schwann cell development and myelination. Semin Cell Dev Biol. 2010;21(9):922‐928. 20832498 10.1016/j.semcdb.2010.08.008 PMC2991617

[116] Taveggia C , Zanazzi G , Petrylak A , et al. Neuregulin‐1 type III determines the ensheathment fate of axons. Neuron. 2005;47:681‐694. 16129398 10.1016/j.neuron.2005.08.017 PMC2387056

[117] Shin YK , Jang SY , Park SY , et al. Grb2‐associated binder‐1 is required for neuregulin‐1‐induced peripheral nerve myelination. J Neurosci. 2014;34:7657‐7662. 24872569 10.1523/JNEUROSCI.4947-13.2014 PMC6795250

[118] Grigoryan T , et al. Wnt/Rspondin/β‐catenin signals control axonal sorting and lineage progression in Schwann cell development. Proc Natl Acad Sci. 2013;110(45):18174‐18179. 24151333 10.1073/pnas.1310490110 PMC3831430

[119] Lewallen KA , Shen YAA , de la Torre AR , Ng BK , Meijer D , Chan JR . Assessing the role of the cadherin/catenin complex at the Schwann cell–axon interface and in the initiation of myelination. J Neurosci. 2011;31:3032‐3043. 21414924 10.1523/JNEUROSCI.4345-10.2011 PMC3758556

[120] Beirowski B . The LKB1‐AMPK and mTORC1 metabolic signaling networks in schwann cells control axon integrity and myelination: assembling and upholding nerves by metabolic signaling in Schwann cells. Bioessays. 2019;41:e1800075. 10.1002/bies.201800075 30537168

[121] Grigoryan T , Birchmeier W . Molecular signaling mechanisms of axon–glia communication in the peripheral nervous system. Bioessays. 2015;37:502‐513. 25707700 10.1002/bies.201400172

[122] Morrison BM , Lee Y , Rothstein JD . Oligodendroglia: metabolic supporters of axons. Trends Cell Biol. 2013;23:644‐651. 23988427 10.1016/j.tcb.2013.07.007 PMC3842360

[123] Baltan S . Can lactate serve as an energy substrate for axons in good times and in bad, in sickness and in health? Metab Brain Dis. 2015;30:25‐30. 25034458 10.1007/s11011-014-9595-3 PMC4297510

[124] Saab AS , Tzvetanova ID , Nave KA . The role of myelin and oligodendrocytes in axonal energy metabolism. Curr Opin Neurobiol. 2013;23:1065‐1072. 24094633 10.1016/j.conb.2013.09.008

[125] Eckersley L . Role of the Schwann cell in diabetic neuropathy. Int Rev Neurobiol. 2002;50:293‐321. 12198814 10.1016/s0074-7742(02)50081-7

[126] Chowdhury SK , Smith DR , Fernyhough P . The role of aberrant mitochondrial bioenergetics in diabetic neuropathy. Neurobiol Dis. 2013;51:56‐65. 22446165 10.1016/j.nbd.2012.03.016

[127] Kim ES , Isoda F , Kurland I , Mobbs CV . Glucose‐induced metabolic memory in Schwann cells: prevention by PPAR agonists. Endocrinology. 2013;154:3054‐3066. 23709088 10.1210/en.2013-1097 PMC5393331

[128] Zenker J , Ziegler D , Chrast R . Novel pathogenic pathways in diabetic neuropathy. Trends Neurosci. 2013;36:439‐449. 23725712 10.1016/j.tins.2013.04.008

[129] Goncalves NP , et al. Schwann cell interactions with axons and microvessels in diabetic neuropathy. Nat Rev Neurol. 2017;13:135‐147. 28134254 10.1038/nrneurol.2016.201 PMC7391875

[130] Dibble CC , Manning BD . Signal integration by mTORC1 coordinates nutrient input with biosynthetic output. Nat Cell Biol. 2013;15:555‐564. 23728461 10.1038/ncb2763 PMC3743096

[131] Laplante M , Sabatini DM . Regulation of mTORC1 and its impact on gene expression at a glance. J Cell Sci. 2013;126:1713‐1719. 23641065 10.1242/jcs.125773 PMC3678406

[132] Wong KM , Babetto E , Beirowski B . Axon degeneration: make the Schwann cell great again. Neural Regen Res. 2017;12:518‐524. 28553320 10.4103/1673-5374.205000 PMC5436338

[133] Beirowski B , Wong KM . mTORC1 promotes Schwann cell cycling and myelinogenesis. Cell Cycle. 2017;16:1637‐1638. 28820327 10.1080/15384101.2017.1360635 PMC5602370

[134] Wong KM , Beirowski B . Multiple lines of inhibitory feedback on AKT kinase in Schwann cells lacking TSC1/2 hint at distinct functions of mTORC1 and AKT in nerve development. Commun Integr Biol. 2018;11:e1433441. 10.1080/19420889.2018.1433441 PMC5824964 29497474

[135] Norrmen C , et al. mTORC1 controls PNS myelination along the mTORC1‐RXRgamma‐SREBP‐lipid biosynthesis axis in Schwann cells. Cell Rep. 2014;9:646‐660. 25310982 10.1016/j.celrep.2014.09.001

[136] Maillet V , Boussetta N , Leclerc J , et al. LKB1 as a gatekeeper of hepatocyte proliferation and genomic integrity during liver regeneration. Cell Rep. 2018;22:1994‐2005. 29466728 10.1016/j.celrep.2018.01.086

[137] Bays JL , Campbell HK , Heidema C , Sebbagh M , DeMali KA . Linking E‐cadherin mechanotransduction to cell metabolism through force‐mediated activation of AMPK. Nat Cell Biol. 2017;19:724‐731. 28553939 10.1038/ncb3537 PMC5494977

[138] Jansen M , ten Klooster JP , Offerhaus GJ , Clevers H . LKB1 and AMPK family signaling: the intimate link between cell polarity and energy metabolism. Physiol Rev. 2009;89:777‐798. 19584313 10.1152/physrev.00026.2008

[139] Beirowski B , Babetto E , Golden JP , et al. Metabolic regulator LKB1 is crucial for Schwann cell‐mediated axon maintenance. Nat Neurosci. 2014;17:1351‐1361. 25195104 10.1038/nn.3809 PMC4494117

[140] Pooya S , Liu X , Kumar VBS , et al. The tumour suppressor LKB1 regulates myelination through mitochondrial metabolism. Nat Commun. 2014;5:4993. 25256100 10.1038/ncomms5993 PMC4431623

[141] Tzvetanova ID , Nave KA . Axons hooked to Schwann cell metabolism. Nat Neurosci. 2014;17:1293‐1295. 25254976 10.1038/nn.3825

[142] Love S . Demyelinating diseases. J Clin Pathol. 2006;59:1151‐1159. 17071802 10.1136/jcp.2005.031195 PMC1860500

[143] Kaufman DM , Geyer HL , Milstein MJ . Kaufman's Clinical Neurology for Psychiatrists. Amsterdam, Netherlands: Elsevier Health Sciences; 2016.

[144] Muheremu A , Ao Q . Past, present, and future of nerve conduits in the treatment of peripheral nerve injury. Biomed Res Int. 2015;2015:1‐6. 10.1155/2015/237507 PMC4600484 26491662

[145] Rodrigues MCO , Rodrigues AA , Glover LE , Voltarelli J , Borlongan CV . Peripheral nerve repair with cultured schwann cells: getting closer to the clinics. Scientific World Journal. 2012;2012:1‐10. 10.1100/2012/413091 PMC3373143 22701355

[146] Duncan I , et al. Transplantation of rat Schwann cells grown in tissue culture into the mouse spinal cord. J Neurol Sci. 1981;49:241‐252. 7217983 10.1016/0022-510x(81)90082-4

[147] Lopatina T , Kalinina N , Karagyaur M , et al. Adipose‐derived stem cells stimulate regeneration of peripheral nerves: BDNF secreted by these cells promotes nerve healing and axon growth de novo. PLoS One. 2011;6:e17899. 21423756 10.1371/journal.pone.0017899 PMC3056777

[148] Kingham PJ , Kalbermatten DF , Mahay D , Armstrong SJ , Wiberg M , Terenghi G . Adipose‐derived stem cells differentiate into a Schwann cell phenotype and promote neurite outgrowth in vitro. Exp Neurol. 2007;207:267‐274. 17761164 10.1016/j.expneurol.2007.06.029

[149] di Summa PG , Kingham PJ , Raffoul W , Wiberg M , Terenghi G , Kalbermatten DF . Adipose‐derived stem cells enhance peripheral nerve regeneration. J Plast Reconstr Aesthet Surg. 2010;63:1544‐1552. 19828391 10.1016/j.bjps.2009.09.012

[150] Xu Y , Liu L , Li Y , et al. Myelin‐forming ability of Schwann cell‐like cells induced from rat adipose‐derived stem cells in vitro. Brain Res. 2008;1239:49‐55. 18804456 10.1016/j.brainres.2008.08.088

[151] Xu Y , Liu Z , Liu L , et al. Neurospheres from rat adipose‐derived stem cells could be induced into functional Schwann cell‐like cells in vitro. BMC Neurosci. 2008;9:21. 18269732 10.1186/1471-2202-9-21 PMC2257969

[152] Jiang L , Zhu JK , Liu XL , Xiang P , Hu J , Yu WH . Differentiation of rat adipose tissue‐derived stem cells into Schwann‐like cells in vitro. Neuroreport. 2008;19:1015‐1019. 18580571 10.1097/WNR.0b013e3283040efc

[153] Heine W , et al. Transplanted neural stem cells promote axonal regeneration through chronically denervated peripheral nerves. Exp Neurol. 2004;189:231‐240. 15380475 10.1016/j.expneurol.2004.06.014

[154] Murakami T , Fujimoto Y , Yasunaga Y , et al. Transplanted neuronal progenitor cells in a peripheral nerve gap promote nerve repair. Brain Res. 2003;974:17‐24. 12742620 10.1016/s0006-8993(03)02539-3

[155] Niapour A , Karamali F , Karbalaie K , et al. Novel method to obtain highly enriched cultures of adult rat Schwann cells. Biotechnol Lett. 2010;32:781‐786. 20213527 10.1007/s10529-010-0230-z

[156] Takahashi K , Tanabe K , Ohnuki M , et al. Induction of pluripotent stem cells from adult human fibroblasts by defined factors. Cell. 2007;131:861‐872. 18035408 10.1016/j.cell.2007.11.019

[157] Ma M‐S , Boddeke E , Copray S . Pluripotent stem cells for Schwann cell engineering. Stem Cell Rev Report. 2015;11:205‐218. 10.1007/s12015-014-9577-1 25433863

[158] Kim H‐S , Lee J , Lee DY , et al. Schwann cell precursors from human pluripotent stem cells as a potential therapeutic target for myelin repair. Stem Cell Rep. 2017;8:1714‐1726. 10.1016/j.stemcr.2017.04.011 PMC5469943 28506533

[159] Lee G , Chambers SM , Tomishima MJ , Studer L . Derivation of neural crest cells from human pluripotent stem cells. Nat Protoc. 2010;5:688‐701. 20360764 10.1038/nprot.2010.35

[160] Tsuji O , Miura K , Okada Y , et al. Therapeutic potential of appropriately evaluated safe‐induced pluripotent stem cells for spinal cord injury. Proc Natl Acad Sci. 2010;107:12704‐12709. 20615974 10.1073/pnas.0910106107 PMC2906548

[161] Wang A , Tang Z , Park IH , et al. Induced pluripotent stem cells for neural tissue engineering. Biomaterials. 2011;32:5023‐5032. 21514663 10.1016/j.biomaterials.2011.03.070 PMC3100451

[162] Harrop JS , Hashimoto R , Norvell D , et al. Evaluation of clinical experience using cell‐based therapies in patients with spinal cord injury: a systematic review. J Neurosurg Spine. 2012;17(Suppl1):230‐246. 22985383 10.3171/2012.5.AOSPINE12115

[163] Krause MP , Dworski S , Feinberg K , et al. Direct genesis of functional rodent and human Schwann cells from skin mesenchymal precursors. Stem Cell Rep. 2014;3:85‐100. 10.1016/j.stemcr.2014.05.011 PMC4110792 25068124

[164] Sakaue M , Sieber‐Blum M . Human epidermal neural crest stem cells as a source of Schwann cells. Development. 2015;142:3188‐3197. 26251357 10.1242/dev.123034 PMC4582175

[165] Ni Y , Zhang K , Liu X , et al. miR‐21 promotes the differentiation of hair follicle‐derived neural crest stem cells into Schwann cells. Neural Regen Res. 2014;9:828‐836. 25206896 10.4103/1673-5374.131599 PMC4146246

[166] Al‐Zer H , et al. Enrichment and schwann cell differentiation of neural crest‐derived dental pulp stem cells. In Vivo. 2015;29:319‐326. 25977377

[167] Coste C , Neirinckx V , Sharma A , et al. Human bone marrow harbors cells with neural crest‐associated characteristics like human adipose and dermis tissues. PLoS One. 2017;12:e0177962. 10.1371/journal.pone.0177962 PMC5500284 28683107

[168] Saulite L , Vavers E , Zvejniece L , Dambrova M , Riekstina U . The differentiation of skin mesenchymal stem cells towards a schwann cell phenotype: impact of sigma‐1 receptor activation. Mol Neurobiol. 2018;55:2840‐2850. 28455697 10.1007/s12035-017-0511-9

[169] Mozafari S , Laterza C , Roussel D , et al. Skin‐derived neural precursors competitively generate functional myelin in adult demyelinated mice. J Clin Invest. 2015;125:3642‐3656. 26301815 10.1172/JCI80437 PMC4588275

[170] Vasudeva VS , Abd‐El‐Barr MM , Chi JH . Implantation of neonatal skin–derived precursor schwann cells improves outcomes after incomplete cervical spinal cord injury in rats. Neurosurgery. 2015;77:N15‐N17. 26181787 10.1227/01.neu.0000467294.10763.69

[171] Shakhbazau A , Mohanty C , Kumar R , Midha R . Sensory recovery after cell therapy in peripheral nerve repair: effects of naive and skin precursor‐derived Schwann cells. J Neurosurg. 2014;121:423‐431. 24949674 10.3171/2014.5.JNS132132

[172] Khuong HT , Kumar R , Senjaya F , et al. Skin derived precursor Schwann cells improve behavioral recovery for acute and delayed nerve repair. Exp Neurol. 2014;254:168‐179. 24440805 10.1016/j.expneurol.2014.01.002

[173] Kang HK , Min SK , Jung SY , et al. The potential of mouse skin‐derived precursors to differentiate into mesenchymal and neural lineages and their application to osteogenic induction in vivo. Int J Mol Med. 2011;28:1001‐1011. 21879252 10.3892/ijmm.2011.785

[174] Walsh S , Biernaskie J , Kemp SWP , Midha R . Supplementation of acellular nerve grafts with skin derived precursor cells promotes peripheral nerve regeneration. Neuroscience. 2009;164:1097‐1107. 19737602 10.1016/j.neuroscience.2009.08.072

[175] Biernaskie J , Sparling JS , Liu J , et al. Skin‐derived precursors generate myelinating Schwann cells that promote remyelination and functional recovery after contusion spinal cord injury. J Neurosci. 2007;27:9545‐9559. 17804616 10.1523/JNEUROSCI.1930-07.2007 PMC6672973

[176] Grochmal J , Dhaliwal S , Stys PK , van Minnen J , Midha R . Skin‐derived precursor schwann cell myelination capacity in focal tibial demyelination. Muscle Nerve. 2014;50:262‐272. 24282080 10.1002/mus.24136

[177] Zhu C , Huang J , Xue C , et al. Skin derived precursor Schwann cell‐generated acellular matrix modified chitosan/silk scaffolds for bridging rat sciatic nerve gap. Neurosci Res. 2018;135:21‐31. 29288689 10.1016/j.neures.2017.12.007

[178] Sparling JS , Bretzner F , Biernaskie J , et al. Schwann cells generated from neonatal skin‐derived precursors or neonatal peripheral nerve improve functional recovery after acute transplantation into the partially injured cervical spinal cord of the rat. J Neurosci. 2015;35:6714‐6730. 25926450 10.1523/JNEUROSCI.1070-14.2015 PMC6605177

[179] Menendez L , Kulik MJ , Page AT , et al. Directed differentiation of human pluripotent cells to neural crest stem cells. Nat Protoc. 2013;8:203‐212. 23288320 10.1038/nprot.2012.156

[180] Tamura Y , Matsumura K , Sano M , et al. Neural crest–derived stem cells migrate and differentiate into cardiomyocytes after myocardial infarction. Arterioscler Thromb Vasc Biol. 2011;31:582‐589. 21212399 10.1161/ATVBAHA.110.214726

