# Supplementary material for: Adult tissue–derived neural crest‐like stem cells: Sources, regulatory networks, and translational potential
Source: Stem Cells Transl Med. 2019 Nov 18;9(3):328–41. doi: 10.1002/sctm.19-0173 (PMC7031649; doi:10.1002/sctm.19-0173)
Supplement: Supplementary file 1 — Figure S1: Adult sources for in vitro expansion of multipotent stem cells exhibiting an NC‐like phenotype. From the above mentioned sources, only cells from dental pulp and hair follicles have been successfully isolated and studied in humans Figure S2: A Neural Crest Stem Cell's journey to myelination. Delaminating, post migratory NC follow Wnt and FGF signals and upregulate key transcription factors like Sox10, FoxD3 and Pax3. By recruiting HDAC1/2, myelin protein 0 is upregulated leading to a Schwann cell precursor phenotype. During this immature Schwann cell stage, Schwann cells initially approach multiple axons and after undergoing significant cytoskeletal reorganization, each one myelinates a single axon, a process referred to as axonal sorting. Finally, the mature Schwann cell envelopes along the neuronal axon with myelin sheath to facilitate saltatory electrical impulse conduction. [file SCT3-9-328-s001.pdf]

Supplemental Information

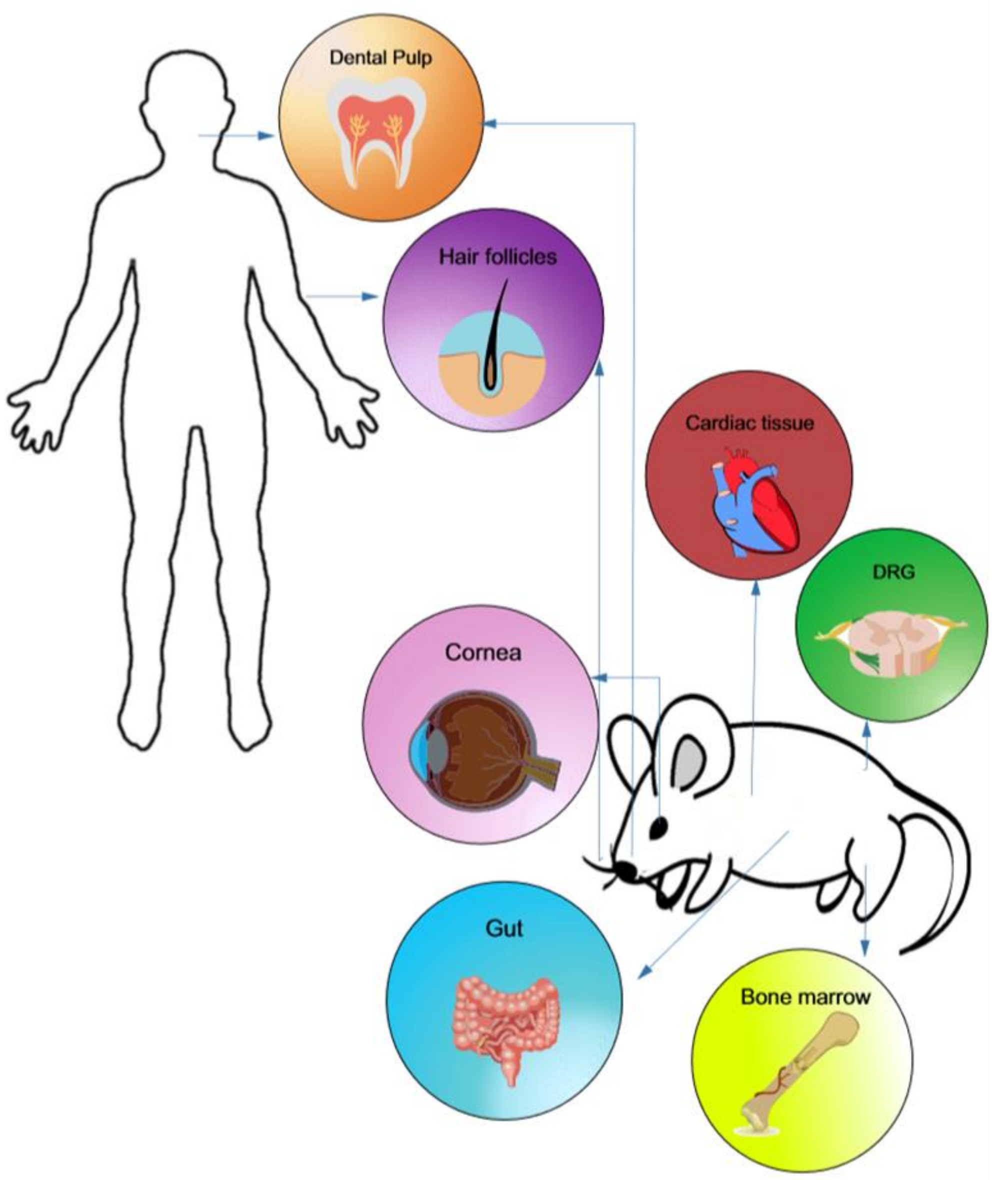

Supplemental Figure S1: *Adult sources for in vitro expansion of multipotent stem cells exhibiting an NC-like phenotype. From the above mentioned sources, only cells from dental pulp and hair follicles have been successfully isolated and studied in humans.*

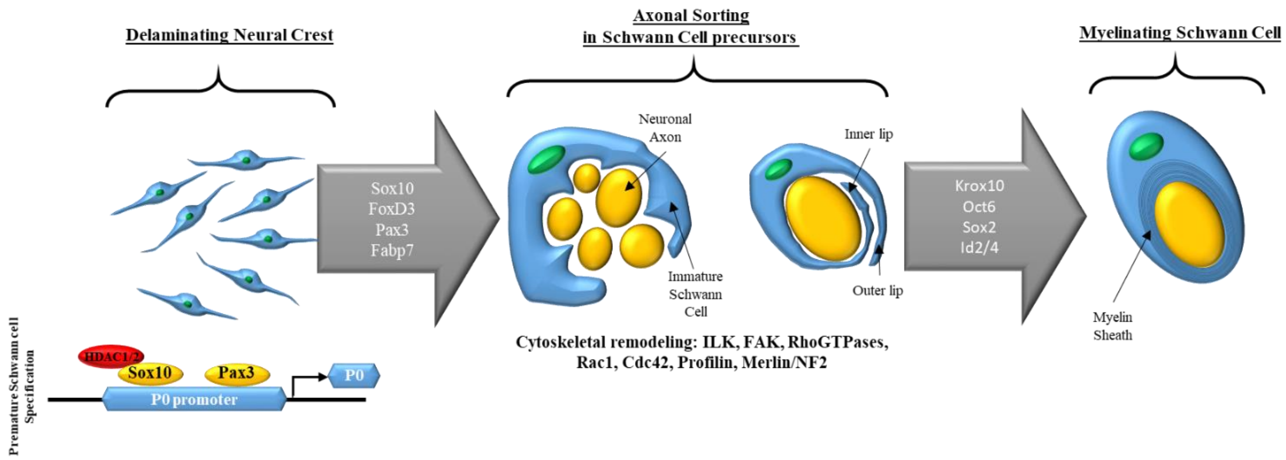

Supplemental Figure S2*: A Neural Crest Stem Cell's journey to myelination. Delaminating, post migratory NC follow Wnt and FGF signals and upregulate key transcription factors like Sox10, FoxD3 and Pax3. By recruiting HDAC1/2, myelin protein 0 is upregulated leading to a Schwann cell precursor phenotype. During this immature Schwann cell stage, Schwann cells initially approach multiple axons and after undergoing significant cytoskeletal reorganization, each one myelinates a single axon, a process referred to as axonal sorting. Finally, the mature Schwann cell envelopes along the neuronal axon with myelin sheath to facilitate saltatory electrical impulse conduction.*
